# The oligosaccharides of Xiasangju alleviates dextran sulfate sodium-induced colitis in mice by inhibiting inflammation

**DOI:** 10.1371/journal.pone.0295324

**Published:** 2023-12-07

**Authors:** Weiguang Sun, Erna Li, Xin Mao, Yulin Zhang, Quxing Wei, Zhiyun Huang, Anfeng Wan, Yuxiao Zou

**Affiliations:** 1 Guangzhou Baiyunshan Xingqun Pharmaceutical Co., Ltd, Guangzhou, 510288, China; 2 Sericultural & Agri-Food Research Institute, Guangdong Academy of Agricultural Sciences/Key Laboratory of Functional Foods, Ministry of Agriculture and Rural Affairs/Guangdong Key Laboratory of Agricultural Products Processing, Guangzhou, 510610, China; University of Tennessee Health Science Center, UNITED STATES

## Abstract

Xiasangju (XSJ) is a traditional Chinese herbal formula consisted of Prunella spica, Mulberry leaf and Chrysanthemi indici flos, which can be used to treat fever, headache and ulcer. To explore the effects of oligosaccharides from XSJ (OX) on colitis, we used dextran sulfate sodium (DSS) to establish colitis mouse models. After administration of OX with different doses on the control and colitis mice, we measured their body weights, disease activity indexes (DAI), lengths and histopathologic changes of colons, spleen indexes. The inflammatory cytokines and oxidative stress-related factors in serum, and the intestinal microbial community in feces were also detected. We found that colitis mice with oral administration of OX showed higher body weights and lower levels of DAI and spleen index. Tissue damages induced by DSS were also alleviated by OX treatment. The colitis mice with OX treatment exhibited lower levels of AST, ALT, BUN, CR, MDA and a down-regulated expression of IL-6 and IL-1β, while the activity of SOD was up-regulated. Furthermore, OX improved the relative abundance of gut microbiota and restored the proportions of Bacteroidetes and Muribaculaceae. We found that oligosaccharides from XSJ alleviated the symptoms of colitis mice through its inhibitory effects on inflammation and oxidative stress, and also regulated the composition of intestinal flora, which indicates a beneficial role for patients with colitis.

## Introduction

Ulcerative colitis (UC) is a chronic inflammatory bowel disease characterized by continuous colon inflammation. Although the etiology of UC is unknown, several factors such as genetic, environmental background and mucosal immune disorders contributing to the disease have been identified [1]. UC patients typically present bloody diarrhea, abdominal pain, urgency, and tenesmus [2]. More than one-third of patients with UC are affected by extraintestinal manifestations or complications beyond the intestinal symptom of the disease, including the liver, lungs, skin, eyes, joints and kidneys [3–5]. In recent years, UC has become a global disease with accelerating incidence in newly industrialized countries with a much high prevalence in males aged 15–30 years old [6,7].

As a chronic inflammatory disease, UC requires a long-term drug treatment such as 5-Aminosalicylates (5-ASA) and corticosteroids [2]. About 40% of patients with UC are stuck with maintenance therapies because of inadequate response to existing medications and the side effects [8,9]. Compared with 5-ASA and corticosteroid therapies, traditional Chinese herbal medicine treatments have showen obvious therapeutic effects on UC with advantages such as promoting mucosal regeneration and relieving inflammation with lower toxicity [10–12].

Xiasangju (XSJ) is a traditional Chinese herbal formula consisted of Prunella spica, Mulberry leaf and Chrysanthemi indici flos. XSJ was derived from a classic formula “Sangju Yin”, which was recorded in “Wenbing Tiaobian” edited by Wu Jutong in Qing dynasty. As a kind of popular herbal tea in southern China, XSJ was listed in Chinese intangible cultural heritage in 2006. XSJ can be used to treat fever, headache, ulcer, boil, high blood pressure, dizziness, tinnitus, and sore throat. In addition to the hepatoprotective and renoprotective properties, XSJ showed activities including antioxidant, anti-tumor, anti-diabetes, which mainly contains sesquiterpenes, flavonoids, phenolic compounds, polysaccharides and oligosaccharides [13]. The polysaccharides and oligosaccharides of XSJ were the main components that contribute to its anti-inflammation activities. In this research, we used dextran sulfate sodium (DSS) to induce colitis in mice to detect the potential effects of oligosaccharides from XSJ (OX) on colitis. To further explore the underlying mechanisms, we also tested the influences of OX on oxidative stress, gut microbiota and inflammatory responses in DSS-induced colitis.

## Materials and methods

### Reagents

Xiasangju (XSJ) extract was obtained from Baiyunshan Xingqun Co., Ltd. (Guangzhou, China). Prepared by alcohol precipitation, the polysaccharides concentration of XSJ extract quantified by the phenol-sulfuric acid method was 80mg/mL. The polysaccharides concentration of XSJ extract was diluted to the 50mg/mL, followed by the addition of 1500U/mL glucanase for enzymolysis at 50°C for 4h and finally got oligosaccharides of XSJ (OX).

### Animals

Male Balb/c mice aged 6 weeks (14–18 g) were purchased from the Medical Laboratory Animal Center of Guangdong Province. All animal procedures were approved by the Institutional Care and Ethical Committee of Guangdong Academy of Agricultural Sciences and conformed to Guide for the Care and Use of Laboratory Animals of the National Institutes of Health. All mice were maintained in a specific pathogen-free facility (SPF) environment with free access to food and water on a 12–12 h light/dark cycle at temperatures of 22–25°C with 60–70% humidity. All research staff holding animal experiment work license granted by Guangdong Laboratory Animal Monitoring Institute.

### Animal treatment protocol

36 male Balb/c mice aged 6 weeks were randomly assigned to 6 experimental groups (*n* = 6): no-treatment control group (NC), model group (MC), positive control group (PC), OX-low dose group (XL), OX-middle dose group (XM), OX-high dose group (XH). The oral gavage (Table 1) lasted for three weeks (day 1 to day 21), and 3.5% DSS (MP Biomedicals, USA) solution replaced their drinking water to establish the colitis model (day 15 to day 21). During the experiment, the body weights, health and behavior of the mice had been recorded every day. The mice were sacrificed on day 22, before which the mice fasted for 12 hours. Mice will be sacrificed if they reached the humane endpoints: loss of >15% body weight, rectal prolapse, or signs of pain (poor grooming and hunched posture). No animal died before meeting criteria for euthanasia.

**Table 1:** Animal treatment protocol.

Groups	Days 1–14	Days 15–21
Normal Control (NC)	0.9% Nacl	0.9% Nacl
Model Control (MC)	0.9% Nacl	0.9% Nacl+ 3.5%DSS
Positive Control (PC)	123.3 mg/kg Berberine	123.3 mg/kg Berberine+ 3.5%DSS
OX-Low dose (XL)	750 mg/kg OX	750 mg/kg OX+ 3.5%DSS
OX-Medium dose (XM)	1500 mg/kg OX	1500mg/kg OX+ 3.5%DSS
OX-High dose (XH)	3000 mg/kg OX	3000 mg/kg OX + 3.5%DSS

The mice were anesthetized with 2% pentobarbital sodium (Qiyun Biotechnology Ltd, Guangzhou, China), peripheral blood were collected from retro-orbital veins, and then euthanized by cervical dislocation (death occurring within 10 seconds). The mice’s spleens were removed and weighed, the lengths of mice’s colon and cecum were isolated and measured. The serum was collected by refrigerated centrifugation of the peripheral blood at 3000r/min for 15 min and stored at -80°C.

### Clinical evaluation of colitis

The body weight, shape changes of stool and fecal blood of the mice were recorded and scored every day during the DDS-induced colitis process (days 15–21) by the DAI (Disease activity index, DAI) detailed in Table 2. The DAI score = average score of weight loss, stool consistency, and bleeding.

**Table 2:** Disease activity index (DAI) score parameters.

Score	Weight loss (%)	Stool consistency	Bloody stool
0	≥0	Normal (Formed)	None
1	0~3	Slightly loose	Slightly bloody
2	-3~-6	Semi-formed stool with mucus	Bloody
3	-6~-9	Loose	Visible traces of blood
4	-9~-12	Water diarrhea	Gross rectal bleeding

### Liver and kidney function tests

The alanine aminotransferase (ALT), aspartate aminotransferase (AST), creatinine (CR) and blood urea nitrogen (BUN) levels in serum samples were tested by commercially available assay kits (Jiancheng Biotechnology Ltd, Nanjing, China) according to the manufacturer’s instructions to quantify the extent of liver and kidney damages and activity changes.

### Enzyme-Linked Immunosorbent Assay (ELISA)

The supernatants of colon tissue homogenates were collected to test the level of IL-6 (Interleukin-6, IL-6) by commercially available ELISA Kits (Xinbosheng, Shenzhen, China) according to the manufacturer’s instructions to quantify the level of inflammatory cytokines production.

### Evaluation of superoxide dismutase (SOD), malondialdehyde equivalents (MDA)

The equivalent activities of SOD and MDA in serum and colon tissue homogenates were detected by commercially available kits (Geruisi, Suzhou, China) according to the manufacturer’s instructions to quantify the level of oxidative stress.

### Immunohistochemistry

The colons were fixed with paraformaldehyde, and then dehydrated and embedded in paraffin for section. Hematoxylin-eosin staining was used to observe the histopathologic changes. The colonic tissue after section was incubated with IL-6 and IL-1βantibody (Servicebio, Wuhan, China), and then incubated with HRP labeled secondary antibody. DAB was used for color development.

### Intestinal flora analysis

The bacterial DNA in mice feces from intestinal contents was extracted and assayed referring to the previously reported method [14]. The amplification universal primers of 16S rDNA gene (V3-V4 regions) were 338F and 806R.

### Statistics

All values are expressed as mean±SEM. Statistical analysis was performed using one-way ANOVA with Dunnett’s multiple comparisons test by SPSS version 21.0 statistical software. Differences were regarded as statistically significant at *P* < 0.05. All data was exhibited by using GraphPad Prism 7 and Origin software for visual.

## Results

### Effects of OX on body weight and DAI of DSS-induced colitis mice

**Fig 1:**
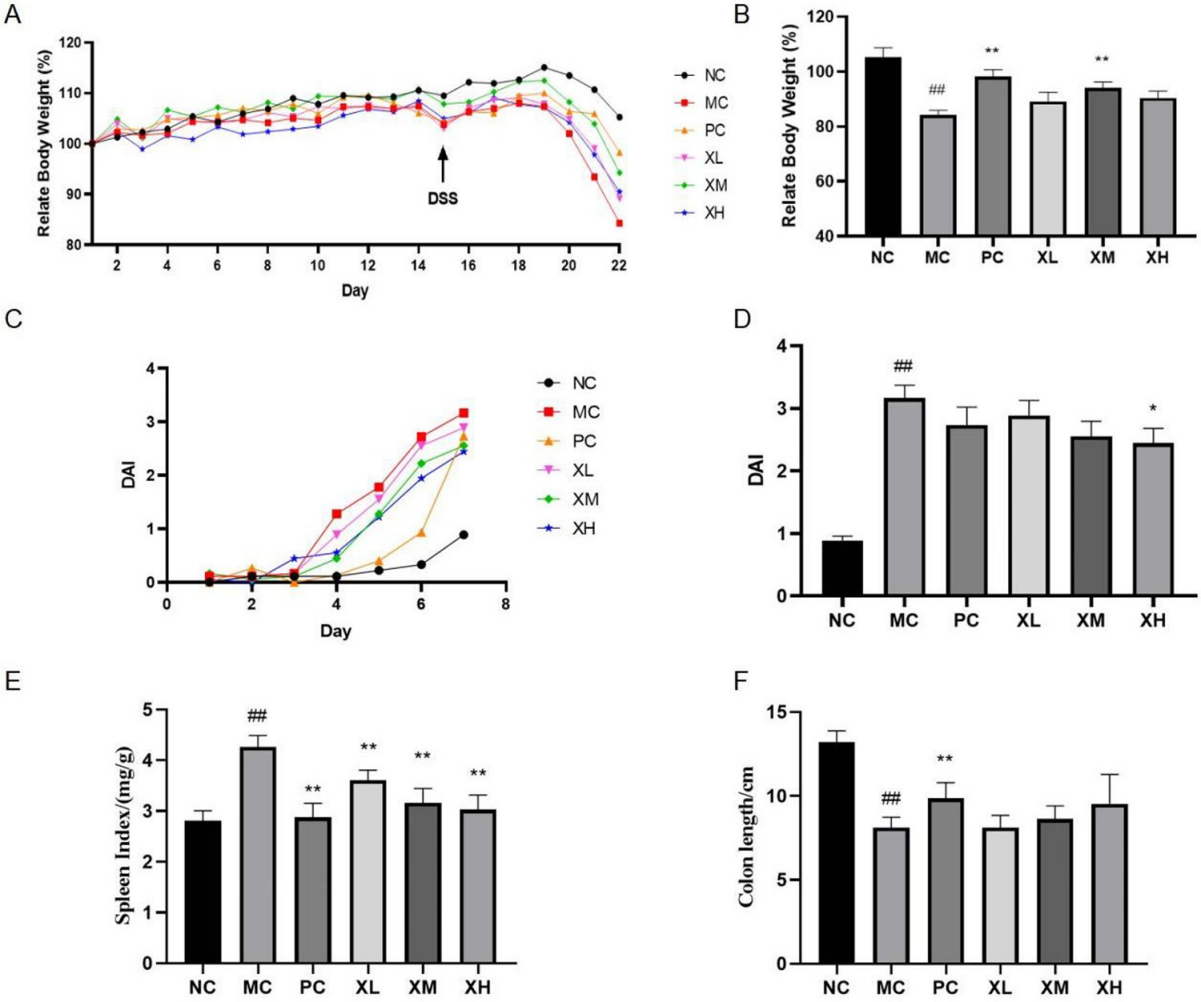
The effect of OX on DSS-induced colitis mice. (A)The changes of relative body weight in different groups from day 1 to day 22. (B) The averages of relative body weight from different groups on day 22. (C) The changes of DAI from different groups in DSS treatment.(D) The averages of DAI from different groups on day 22. (E) The changes of the spleen index from different groups on day 22. (F) The changes of the colon length from different groups on day 22. NC: Normal control group; MC: Model control group; PC: Berberine 123.3mg/kg; XL: OX 750 mg/kg; XM: OX 1500 mg/kg; XH: OX 3000 mg/kg. Compared with NC: ^##^*P* < 0.01; compared with MC: *P < 0.05, **P < 0.01.

The body weight of mice was reduced by DSS (Fig 1A). Compared with the NC group, the average of relative body weight (ratios of body weight to that of day 1) of MC group decreased from 105.26% to 84.29% on day 22 (*P*<0.01) (Fig 1B). The relative body weight of XM group (94.26%) was significantly higher than that of MC group (*P*<0.01) (Fig 1B), indicating an alleviatory effect for the weight loss caused by DSS.

Disease activity index (DAI) was used to evaluate the severity of colitis by body weight loss, stool consistency alteration, and bloody stools. On the fourth day of the DSS-inducing process, the MC group appeared loose and bloody stools. The DAI score of MC group was as high as 3.17 on day 22, which was inhibited by the administration of 3000 mg/kg of OX (*P*<0.05) (Fig 1C and 1D).

### Effects of OX on spleen index and colon length of DSS-induced colitis mice

In UC mice, the increase in spleen weight is a result of intestinal inflammation and anemia. The average of spleen index (ratios of spleen weight to body weight) of MC group was 4.27 mg/g after the DSS-inducing process, which was significantly higher than that of NC group (*P*<0.01) (Fig 1E). The spleen indexes of OX administration groups were effectively inhibited to 3.03–3.60 mg/g (*P*<0.01) (Fig 1E).

Hyperemia and edema contribute to the thinning of intestinal wall and the shortening of colon length in UC mice. With the DSS treatment, the length of colon in MC group was shorter than NC group (*P*<0.01), while the treatment of OX might slightly inhibt this pathological process (*P*>0.05) (Fig 1F).

### Effects of OX on liver and kidney in DSS-induced colitis mice

**Fig 2:**
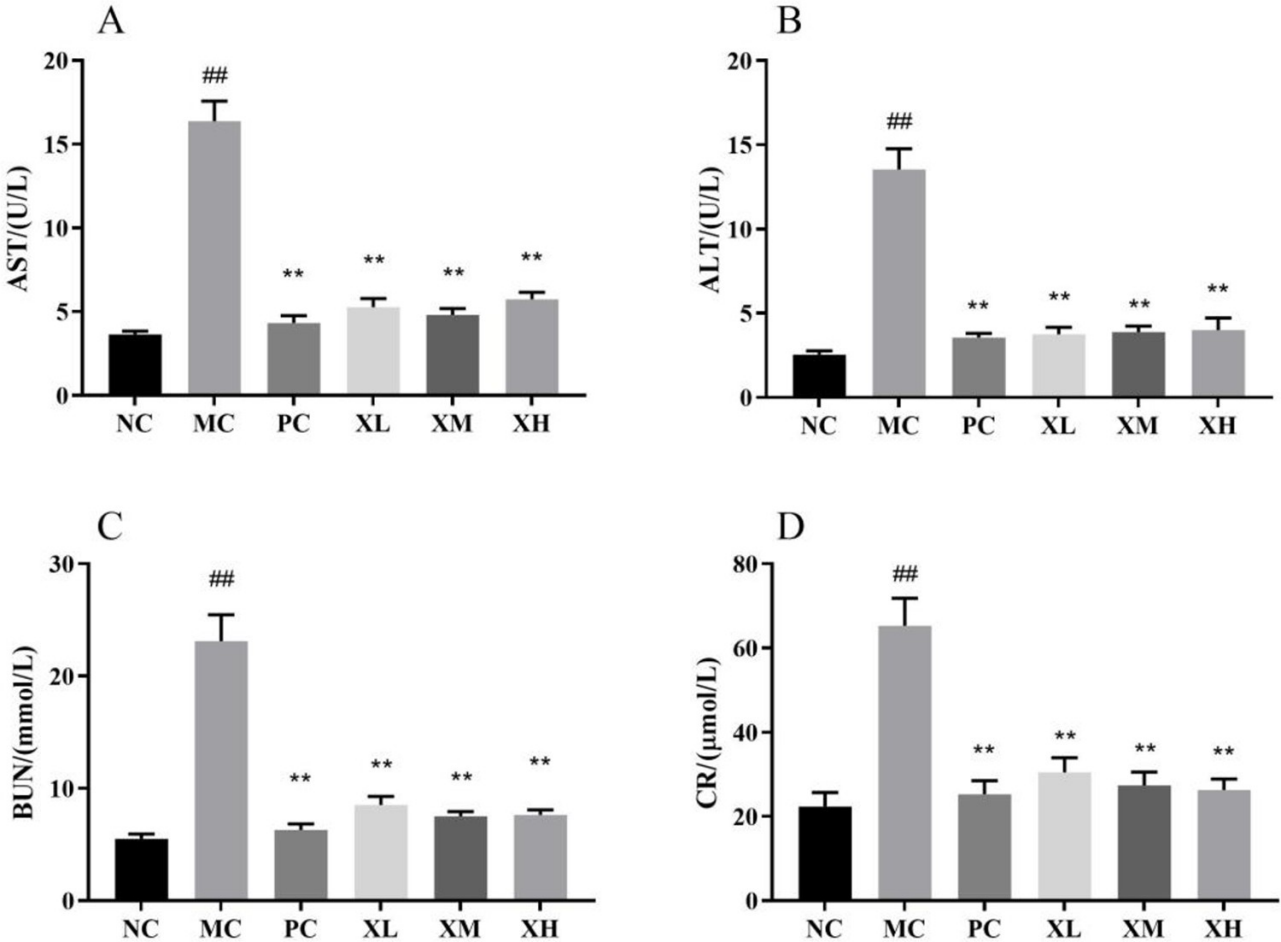
Effects of OX on liver and kidney functions of DSS-induced colitis mice. (A-D) The average levels of serum AST (A), ALT (B), BUN (C) and CR (D) in different groups. NC: Normal control group; MC: Model control group; PC: Berberine 123.3mg/kg; XL: OX 750 mg/kg; XM: OX 1500 mg/kg; XH: OX 3000 mg/kg. Compared with NC: ^##^p < 0.01; compared with MC: **p < 0.01.

Liver and kidney damages are the two main symptoms of UC mice. Aspartate aminotransferase (AST) and alanine aminotransferase (ALT) are important biochemical indicators to quantify liver function. The levels of Blood urea nitrogen (BUN) and creatinine (CR) can be used to evaluate kidney function. The serum levels of AST, ALT, BUN and CR in MC group were much higher than those in NC group (*P*<0.01) (Fig 2), indicating severe liver and kidney damages in UC mice. With the administration of OX, the high levels of AST, ALT, BUN and CR were effectively decreased (*P*<0.01) (Fig 2).

### Effects of OX on oxidative stress of DSS-induced colitis mice

**Fig 3:**
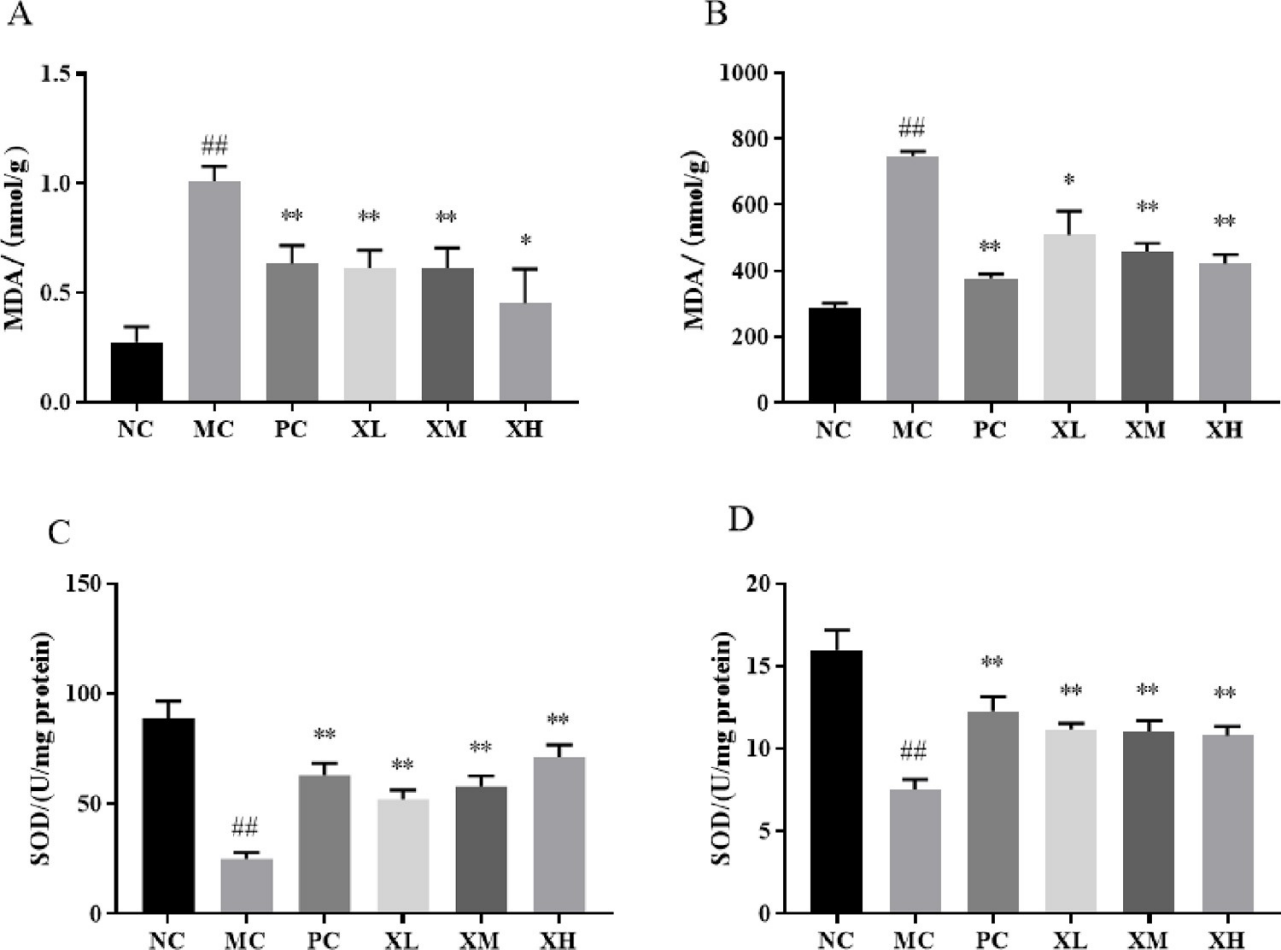
The effect of OX on oxidative stress of DSS-induced colitis mice. (A, B) The average levels of MDA in the serums (A) and colon tissues (B) from different groups. (C, D)The average levels of SOD in the serums (C) and colon tissues (D) from different groups. NC: Normal control group; MC: Model control group; PC: Berberine 123.3mg/kg; XL: OX 750 mg/kg; XM: OX 1500 mg/kg; XH: OX 3000 mg/kg. Compared with NC: ^##^p < 0.01; compared with MC: **p < 0.01, *p < 0.05.

Both malondialdehyde equivalents (MDA) and superoxide dismutase (SOD) play an important role in maintaining balance in the oxidant/anti-oxidant mechanisms. Compared with the NC group, the MDA levels in serums and colon tissues from MC group were much higher (*P*<0.01), which were inhibited by the treatment of OX (*P*<0.05, *P*<0.01) (Fig 3A and 3B). Meanwhile, the lower SOD levels in serums and colon tissues from MC group (*P*<0.01) were improved by the treatment of OX (*P*<0.01) (Fig 3C and 3D).

### Effects of OX on histopathological changes and inflammatory cytokine in colons of DSS-induced colitis mice

**Fig 4:**
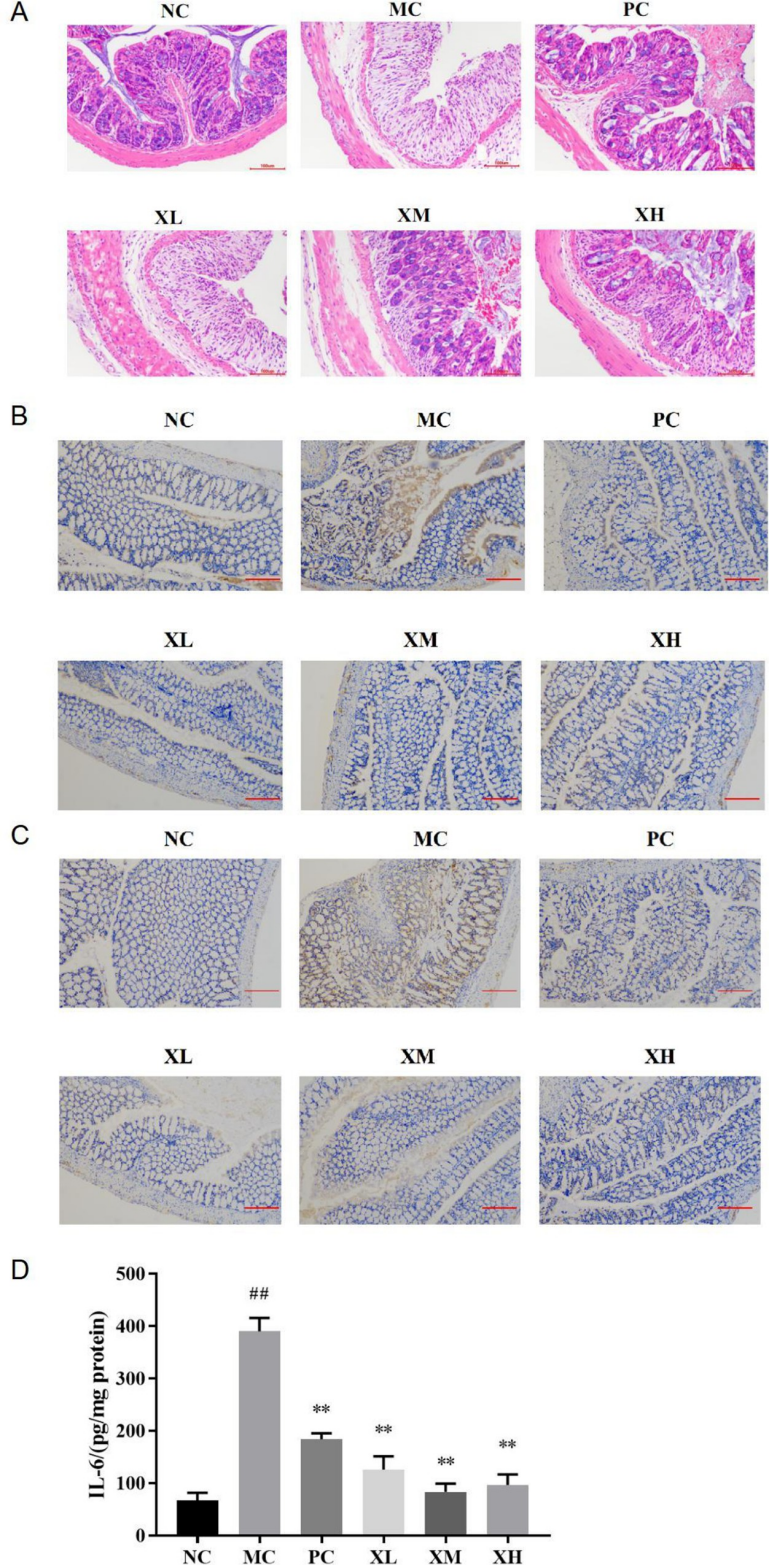
The effect of OX on colon tissues of DSS-induced colitis mice. The histopathological changes of colitis mice. (B) The change of IL-6 (brown) in colons of colitis mice. (C) The change of IL-1β (brown) in colons of colitis mice. (D) The quantitative change of IL-6 level in colons of colitis mice. NC: Normal control group; MC: Model control group; PC: Berberine 123.3mg/kg; XL: OX 750 mg/kg; XM: OX 1500 mg/kg; XH: OX 3000 mg/kg. Scale bar 100 μm (A), Scale bar 200 μm (B, C). Compared with NC: ^##^*P* < 0.01; compared with MC: **P < 0.01.

The UC mice suffer from pathological damage of the colon epithelium, which is associated with high levels of the inflammatory cytokines [15]. The pathological changes of colon tissues of mice in each group were analyzed by HE staining. The NC group demonstrated complete and vivid colonic mucosal epithelial structure and orderly glands without inflammatory cell infiltration (Fig 4A). The MC group demonstrated severe disruption in the colonic and mucosal structures with distortion of crypt structure, loss of goblet cells, and massive inflammatory cell infiltration (Fig 4A). The XM group showed some intact tissue structures and decreased inflammatory infiltration, and the XH group showed a slight injury in colon tissues with intact villi, no mucosal erosion and less inflammatory infiltration (Fig 4A), suggesting an alleviatory effect of OX for the pathological changes in colon tissues.

Furthermore, the expressions of IL-6 and IL-1β in mouse colon tissues were detected by immunohistochemistry (Fig 4B and 4C). After DSS treatment, both IL-6 and IL-1β were highly expressed in the surface of colonic mucosa. After administration of OX, the tissues had correspondingly low levels of IL-6 and IL-1β, indicating that OX inhibited the development of intestinal inflammation.

The level of Interleukin-6 (IL-6) in mice colon was raised from 66.66 to 389.81 pg/mg protein after the DSS treatment (*P*<0.01), which was inhibited to 82.99–125.49 pg/mg protein by the administration of OX (*P*<0.01) (Fig 4D).

### Effects of OX on the intestinal flora in mice with DSS-induced colitis

**Fig 5:**
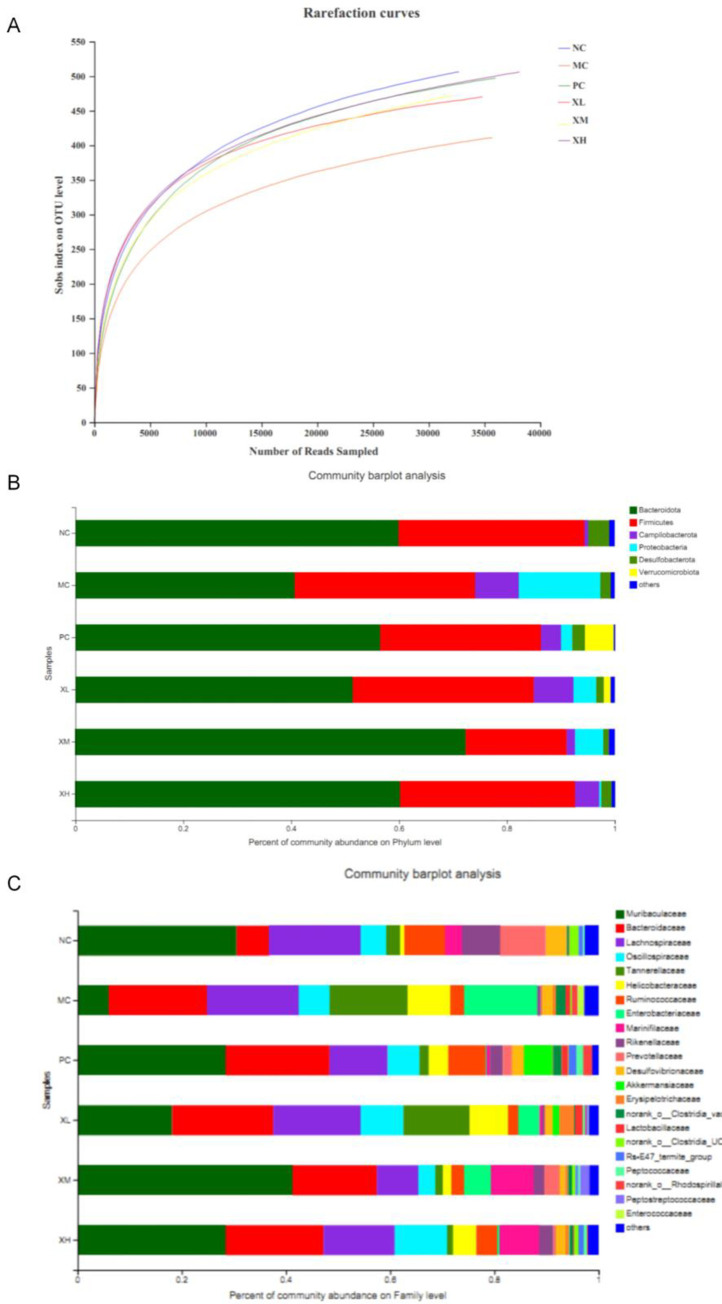
The status of intestinal flora. (A)The graph of rarefaction curves. (B) Distribution of gut microbiota at the taxonomic level of phyla. (C) Distribution of intestinal flora at the taxonomic level of family. NC: Normal control group; MC: Model control group; PC: Berberine 123.3mg/kg; XL: OX 750 mg/kg; XM: OX 1500 mg/kg; XH: OX 3000 mg/kg.

The rarefaction curve is a technique applied to assess the species richness in samples with different sequencing data. As shown in Fig 5, the rarefaction curve of the species ultimately tends to be smooth, indicating that the sequencing data of the sample is reasonable. The sobs index on OTU level in the MC group was lower than that in the NC group (Fig 5A), which indicated a decrease in intestinal flora richness. However, the XH, XM and XL groups exhibited increased intestinal flora richness compared to the MC group (Fig 5A).

Bacteroides, identified as beneficial organisms, are involved in many essential metabolic activities in the colon, including the fermentation of carbohydrates, the utilization of nitrogenous substances, and the bio-transformation of bile acids and other steroids [16]. As showed in Fig 5B, Bacteroidetes accounted for the maximum proportion in each group. The DSS treatment reduced the community abundance of Bacteroidetes. After treatment with OX, the community abundance of Bacteroidetes recovered to varying degrees (Fig 5B), which indicated that the intake of OX benefits the restoration of gut microbiota.

The family classification of the mouse gut microbiota was shown in Fig 5C. Muribaculaceae, Rikenbacteriaceae and Bacteroidetes can degrade complex carbohydrates and are the utilizers of mucin monosaccharides. The DSS inhibited the community abundance of both Muribaculaceae and Rikenbacteriaceae families (Fig 5C). Oral administration of medium and high doses of OX improved the abundance of Muribaculaceae and Rikenbacteriaceae families (Fig 5C). Compared with the NC group, the proportion of Bacteroidetes improved after the oral administration of OX (Fig 5C).

## Disscusion

Oligosaccharides are carbohydrates that are composed of 2–10 monosaccharide units [17]. Non-digestible oligosaccharides have recently been interested as prebiotics by more and more researchers. Oligosaccharides have been considered to associate with numerous health benefits through the modulation of intestinal microbiota [18]. Fructo-oligosaccharide and oligosaccharides from *Amorphophallus konjac* and *Cichorium intybus* were proven to release colitis symptoms in the murine model through the regulation of oxidative stress, inflammation and fecal microbiota [19–21]. OX is developed from XSJ, a classic anti-inflammatory Chinese formula. In this research, we tested the effects of OX on DSS-induced colitis and investigated its underlying mechanisms through the evaluation of inflammatory response, oxidative stress-related indicators and intestinal microbial composition.

DSS-induced colitis mainly results in intestinal tract disorders, such as pathological damage, shortened colon length and abnormal stools. DAI has derived from the typical clinical symptoms of UC such as weight loss, diarrhea and rectal bleeding [22]. The treatment of OX mitigated weight loss, DAI increase, colon shortening and pathological damage in colitis mice (Figs 1 and 4), which suggests that the OX can prevent colon injury in colitis. Apart from gastrointestinal tract illness, UC patients show symptoms of other organs [23]. Spleen is the most important immune organ, and patients with colitis may experience splenomegaly. Previous research showed that inflammatory bowel disease results in acute kidney injury and inflammation in the liver [24,25]. The oral administration of OX reversed the increase of spleen index and biomarkers of liver and kidney functions (Figs 1 and 2), indicating that OX effectively limit the development of colitis complications.

In inflammation bowel disease, disruption of the gut mucosal activates the innate immune system and results in the secretion of reactive oxygen species (ROS) and pro-inflammatory mediators. The expression of ROS may contribute to the elimination of inflammation. However, the over-production of ROS will cause tissue injury [26]. SOD and MDA are classic markers of anti-oxidant and oxidant in oxidative stress response [27]. Our results showed that DSS-mediated oxidative stress induced the increase of MDA level and the decrease of SOD activity (Fig 3). The treatment of OX effectively inhibited the expression of MDA and improved the SOD activity to reduce oxidative damage (Fig 3). IL-6 is a pro-inflammatory cytokine secreted by monocytes and macrophages and contributes to the restoration of damaged tissues. Whereas, uncontrolled production of IL-6 however contributes to the development of various inflammatory diseases [28]. Our data showed that the treatment of OX effectively reduced the excessive expression of IL-6 and IL-1βinduced by DSS treatment (Fig 4B–4D). Thus, the OX played a protective role in inflammation and oxidative damage of DSS-induced colitis in mice.

More and more evidence proved that intestinal microflora plays a prominent role in the development of UC. Diversion of the fecal stream is recognized to result in the remission of colitis, which suggests that optimizing intestinal microbiota is an alternative strategy for UC treatment [29]. The treatment of OX reversed the down-regulation of the gut microbiome richness in DSS-induced colitis mice (Fig 5A). Meanwhile, the proportions of Muribaculaceae and Rikenbacteriaceae families and the Bacteroidetes were restored after treatment with OX (Fig 5B and 5C), which indicates that OX have a recovery effect on the intestinal flora.

In summary, our data demonstrate the potential of OX in the prevention of DSS-induced colitis. The OX relieved clinical symptoms of the colitis, and furthermore, the medium dose (1500mg/kg/d) and high dose (3000mg/kg/d) of OX suggest a promising anti-inflammatory therapeutic strategy for colitis.
